# Genetic divergence in natural and farm populations of Pengba fish, *Osteobrama belangeri* (Valenciennes, 1844), an endemic fish of North-East India derived from mtDNA *ATPase 6/8* gene

**DOI:** 10.1080/23802359.2017.1372700

**Published:** 2017-09-12

**Authors:** Manorama Maisnam, Arti Gupta, Kuldeep K. Lal, Rajeev K. Singh, Vindhya Mohindra

**Affiliations:** ICAR-National Bureau of Fish Genetic Resources, Lucknow, India

**Keywords:** *Osteobrama belangeri*, North-East India, ATPase 6/8, mtDNA

## Abstract

Pengba fish, *Osteobrama belangeri* is a freshwater inhabitant, highly endemic, threatened and economically important minor carp for its food value. In the present investigation, population structure of *O. belangeri* was examined using mitochondrial *ATPase 6/8* gene from geographically distinct locations along the Indo-Burma biodiversity hotspot. A total of 80 individuals were collected belonging to natural and farm populations. The hierarchical analysis of molecular variance and conventional Fst values (0.825 in *ATPase 6/8*, *p* < .05) indicated significant genetic structure among populations. The result showed that *ATPase 6/8* genes are potential marker in determining the genetic divergence between natural and farm populations of *O. belangeri* from North-East India.

## Introduction

*Osteobrama belangeri* (Valenciennes 1844), commonly known as Pengba, is highly endemic average size carp with restricted distribution range in India and Myanmar (Vishwanath and Shantakumar 2007). Earlier, this species was plenty in rivers and lakes of Manipur, but the construction of Ithai barrage on the Imphal river resulted to loss of natural habitat which prevented spawning migration of the fish from Chindwin river of Myanmar to the upstream of Imphal river of Manipur during monsoon season (Behera et al. 2009). The anthropogenic activities such as construction of dam, habitat loss and introduction of exotic species which act as major threats are responsible for declined population of *O. belangeri* in Manipur and led to large impact on their natural abundance. During the last three decades, the natural population of *O. belangeri* has decreased remarkably in the North-East region of India and therefore, currently assessed as ‘Near Threatened’ (Vishwanath 2010). In Myanmar, this fish is available in rivers and locally known as ‘Nga-hpeh-oung’ or ‘Nga-net-hua’ (Basudha and Vishwanath 1999).

Mitochondrial DNA (mtDNA) marker has been used increasingly for quantifying levels of genetic differentiation among populations (Tagliavini et al. 2007), detecting barriers to gene flow (Magoulas et al. 2006), and for identifying evolutionary lineages within the species (Bernatchez et al. 1992). *ATPase 6/8* gene, a potential marker analysed for both phylogeny and phylogeography has been found to be highly variable in vertebrates (Zardoya et al. 1996; Yan et al. 2009), and have been analysed successfully in several fish species (Chow and Ushiama 2004; Dammannagoda et al. 2008; Hurwood et al. 2008; Vergara-Chen et al. 2009).

In the recent past, Singh et al. (2014) made a study in genetic stock structure of *O. belangeri* in Indian region and showed no significant genetic differentiation among the sampling localities based on ATPase 6/8 region. In view of gaining importance of mtDNA marker, the aim of the present study is to analyse complete sequences of ATPase 6/8 collected from four different locations namely, Moreh, Tamu, Hiyangthang, and Kodompokpi to determine genetic variation and evaluate their potential in ascertaining genetic differentiation in natural and farm populations of *O. belangeri*.

## Materials and methods

**Figure 1.**
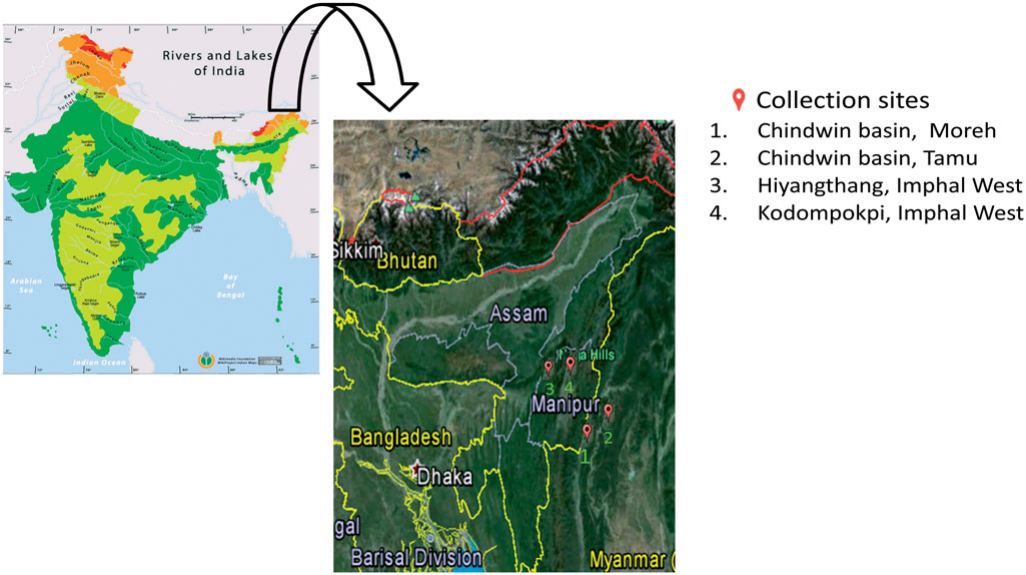
Map showing sample collection sites of *Osteobrama belangeri* (balloon) during present study.

Altogether 80 specimens of *O. belangeri* were obtained from Moreh (24°13′ N, 94°18′ E) and Tamu (24°13′ N, 94°19′ E), a part of Chindwin basin and Hiyangthang (24°42′ N, 93°53′ E) and Kodompokpi (24°44′ N, 93°52′ E) belonging to different geographical sites in Myanmar and India (Figure 1). These specimens were collected from commercial riverine catches with the help of local fishermen and transferred to the laboratory for further studies. Tissue sample was collected from dorsal fin region of the fish and fixed in 95% ethanol and stored at 4 °C until use.

The total genomic DNA was extracted from muscle tissue excised from each fish specimen using phenol–chloroform method (Ruzzante et al. 1996). *ATPase 6/8* gene of mtDNA of 842 bp length was amplified using primers, ATP8.2 L8331 and CO111.2H9236 (Sivasundar et al. 2001). Amplifications were carried out in a 50 μl reaction mixtures containing 1× PCR buffer, 15 mM MgCl_2_, 10 mM of each dNTPs, 5 pmol of each primer, 3 U Taq DNA polymerase and 50–100 ng template DNA. Polymerase chain reactions (PCR) were performed using Peqstar Universal Gradient (Peqlab) with initial denaturation at 94 °C for 5 min, 30 cycles consisting of denaturation at 94 °C for 30 s, annealing at 50 °C for 1 min and extension at 72 °C for 1 min 30 s and final extension at 72 °C for 10 min. The amplified PCR products were checked through 2% agarose gel and were sequenced bidirectionally, to check the validity of the sequence data, using an automated capillary sequencer (ABI 3730Xl) following the manufacturer’s recommendations.

Amplified *ATPase 6/8* genes were sequenced in both the directions to check the validity of the sequence data and DNA sequences were aligned using Multiple Sequence Alignment ClustalW (Thompson et al. 1994). All the sequences of *O. belangeri* of *ATPase 6/8* gene were deposited in Genbank with the accession number KU901618 and KU901697. MEGA 4.1 (Tamura et al. 2007) was used to estimate parameters such as variable sites, conserved sites and nucleotide composition. Haplotype diversity (*h*) and nucleotide diversity (π) were estimated using DnaSP 5.10 (Rozas et al. 2003). Analysis of molecular variance (AMOVA) and Fst values were calculated using Arlequin 3.11 (Excoffier et al. 2005).

## Results

Out of a total of 842 bp of mitochondrial region amplified, there was an overlapping region of 7 bp in between two genes from 159 to 165 bp. The two fragments were analysed together to determine genetic variation in *O. belangeri*. The average frequencies of four nucleotides for all the samples of *O. belangeri* were A: 33.9%; T: 28.6%; C: 25.6%, and G: 12.0%. Nucleotide sequences of ATPase 6/8 were A + T rich (62.5%). It was observed that out of 842 bases, conserved sites, variables sites, parsimony informative sites and singleton were 838, 4, 2, and 2, respectively. Out of the five haplotypes obtained, h01 and h04 were found in samples from Moreh and Tamu; h02 and h03 in Moreh and h04 in Hiyangthang and Kodompokpi. The mean haplotype diversity (*h*) for samples collected from four populations was 0.574, while nucleotide diversity (π) obtained was 0.00077. AMOVA revealed that 82.55% was attributed to variation among population and 17.45% was due to within the population. Fst value was found to be significant at 0.825 (*p* < .05). Population pair wise Fst values ranged from 0.0 to 0.870 (Table 1). Total shared haplotypes were three; haplotypes h01 and h04 were shared between river Moreh and Tamu and haplotype 05 was shared between farm Hiyangthang and Kodompokpi. Moreh had four haplotypes, Tamu had two haplotypes, and one haplotype were observed each in Hiyangthang and Kodompokpi.

**Table 1.** Population pair wise Fst (below diagonal), population specific Fst (at diagonal) and *p* values (above diagonal) between *Osteobrama belangeri* samples collected from natural and farm populations based on ATPase 6/8 sequence.

River/farm	Moreh	Tamu	Hiyangthang	Kodompokpi
Moreh	**0.816**	0.523	0.000	0.000
Tamu	0.003	**0.818**	0.000	0.000
Hiyangthang	0.870*	0.883*	**0.834**	0.991
Kodompokpi	0.870*	0.883*	0.000	**0.834**

Bold values indicate population specific Fst.

* *p* < .05.

## Discussion

Identification of population structure and genetic variation in any species yield substantial information required for effective planning and developing conservation strategies and its application in fisheries management. The results from the present study revealed significant genetic differentiation exists in *O. belangeri* populated in Moreh, Tamu, Hiyangthang, and Kodompokpi based on the analysis of 842 bp ATPase 6/8 sequences.

*O. belangeri* populations exhibited low nucleotide diversity and high haplotype diversity which is consistent with the study carried out by Singh et al. (2014). It suggests that the *O. belangeri* populations experienced founder event and later on, the populations which were fragmented might have undergone rapid expansion followed by formation of new haplotypes found in low frequencies (Grant and Bowen 1998). This is further supported with that of the result as no haplotype was found shared between any populations of natural and farm, which indicated significant genetic separation between these populations.

The result obtained from AMOVA is consistent with significant pairwise and overall Fst values indicating the presence of genetic divergence between populations of Moreh, Tamu, Hiyangthang, and Kodompokpi. The genetic variation among populations (82.55%) observed in this study is higher than that reported (32.4%) for non-migratory species (Vrijenhoek 1998). It can be postulated that high genetic differentiation of *O. belangeri* is due to restricted distribution of the species which subsequently leads to limited gene flow between natural and farm populations. Another possibility is that the fish has been under culture for the last three decades in Indian region and contributes to reproductive isolation of the species.

In recent past, *O. belangeri* was abundant in the Imphal river which drained the adjoining plain area and finally formed the river Manipur, which flows directly from southern part of Manipur into the major river basin Chindwin in northern Myanmar. The migration of *O. belangeri* from Chindwin river to Imphal river was enabled due to the construction of Ithai Barrage in the middle of Imphal river course, the main route for entering into the Indian province. This gives rise to total disconnection of short distance migration of *O. belangeri* during breeding season between the two rivers system which can also be the reason for such genetic variation. Findings on mtDNA support presence of two genetic stocks, river and farm fish and it requires important management program for this species.

In conclusion, ATPase 6/8 region has proven its ability to detect population sub-structure in *O. belangeri*, an important fish having food value and threatened species. It must be taken into account that genetic divergence obtained in farm and wild population could be attributed to the environmental condition of the culture fish. This study will be useful in selecting the fish stocks that maintain better genetic diversity for being used in planning conservation and management strategies of *O. belangeri*.
